# Supplementary material for: The Scorpionfly (Panorpa cognata) Genome Highlights Conserved and Derived Features of the Peculiar Dipteran X Chromosome
Source: Mol Biol Evol. 2023 Nov 21;40(12):msad245. doi: 10.1093/molbev/msad245 (PMC10715201; doi:10.1093/molbev/msad245)
Supplement: msad245_Supplementary_Data [file msad245_supplementary_data.pdf]

Supplementary Material

SUPPLEMENTARY DATASETS 2

Dataset S1: Genome assembly 2

Dataset S2. Transcriptome assembly and transcripts genomic location 2

Dataset S3: Identification of X-linked scaffolds 2

Dataset S4: Gene expression and dosage compensation 2

SUPPLEMENTARY METHODS 3

Methods S1: Homology of *Drosophila melanogaster* to *Panorpa cognata* 3

Methods S2: X-linked genes in *Blatella germanica* and homology to *Panorpa cognata* 3

SUPPLEMENTARY FIGURES 4

Figure S1: BUSCO assessments of our genome and transcriptome assemblies. 4

Figure S2: Male and female genome coverage for all scaffolds. X-linked scaffolds are shown with arrows. 5

Figure S3: Homology of the X chromosomes of *P. cognata* (order: Mecoptera) and three other insects: two Diptera, *C. hominivorax* (suborder: Brachycera) and *N. appendiculata* (suborder: Nematocera), and *L. migratoria* (order: Orthoptera). 6

Figure S4: Percentage of genes on each of the *D. melanogaster* Muller elements that are X-linked in *P. cognata.* 6

Figure S5: Percentage of X-linked and autosomal *P. cognata* genes that are X-linked in *B. germanica* (order: Blattodea). 7

Figure S6: Percentage of X-linked and autosomal *P. cognata* genes that are X-linked in *L. migratoria* (order: Orthoptera). 8

Figure S7: Spearman correlation heatmap displaying the overall similarity in expression profiles of the RNA-seq samples. 8

Figure S8: Dosage compensation in carcasses. 9

Figure S9: Expression level of gene *Painting Of Fourth* (*POF*) in each RNA-seq sample. 10

Figure S10: Detailed per-scaffold nucleotide diversity, GC and repeat content. 12

SUPPLEMENTARY TABLES 13

Table S1: The classification and distribution of repetitive content on the autosomes and the X chromosome. 13

Table S2: List of RNA and DNA samples generated and the analysis they were used for. **13**

Table S3: Statistics for the different assembly steps of the *Panorpa cognata* genome. **13**

Supplementary Datasets

Dataset S1: Genome assembly
Dataset S2. Transcriptome assembly and transcripts genomic location
Dataset S3: Identification of X-linked scaffolds
Dataset S4: Gene expression and dosage compensation
Dataset S5: GC content and nucleotide diversity
Dataset S6: Repeat Content
Dataset S7: Homology (and orthofinder)

All supplementary datasets are available at:
https://seafile.ist.ac.at/d/efa3989c33024b859c02/

Supplementary Methods

Methods S1: Homology of *Drosophila melanogaster* to *Panorpa cognata*

Drosophila melanogaster (order: Diptera; suborder: Brachycera) protein sequences were retrieved from Ensembl (https://ftp.ensembl.org/pub/release-108/fasta/drosophila_melanogaster/pep/). We filtered for the longest sequence of each protein using a Perl script (GetLongestCDS_v2.pl). We used the *Drosophila melanogaster* gene names and their Muller element location according to the CDS from (https://ftp.ensembl.org/pub/release-108/fasta/drosophila_melanogaster/cds/). We used Orthofinder (Emms and Kelly 2019) to obtain the 1-to1 orthologs of *D. melanogaster* and *P. cognata* using the same pipeline as explained in our paper in the "***Homology of the Panorpa cognata and Cochliomyia hominivorax X chromosomes***" methods section.

Methods S2: X-linked genes in *Blattella germanica* and homology to *Panorpa cognata*

We used published *Blattella germanica* (order: Blattodea) DNA reads from two sources: (1) male reads (SRR1566154, SRR1566155, SRR1566159) and one female reads (SRR1566152) from Meisel et al. (2019); and (2) two male reads (SRR9160163, SRR9160164) from NCBI bioproject number PRJNA545466. The female reads from the same bioproject seemed incorrectly labelled and therefore were not used in our analysis. We mapped the reads to the *Blatella germanica* genome (Meisel et al. 2019) using Bowtie2 (version 2/2.4.5 (Langmead and Salzberg 2012)) and then we used SOAP.coverage (version 2.7.7; https://github.com/gigascience/bgi-soap2/tree/mas-ter/tools/soap.coverage) to calculate the coverage depth. As we had five male reads in total, their coverage depth was summed up. We only kept the scaffolds with length greater than 1000 bp. Finally, we calculated the $\log_2$ of the ratio of male to female coverage ($\mathrm{Log_2}$(Male/Female coverage)) for each scaffold. The scaffolds were assigned as X-linked if that ratio was less than the [median($\mathrm{Log_2}$(Male/Female coverage))-0.5], otherwise they were assigned as autosomal. In order to assess the homology of *B. germanica* and *P. cognata*, we used the same pipeline as explained in our paper in the "***Conservation of X-linked gene content between P. cognata and other insects***" methods section.

Supplementary Figures

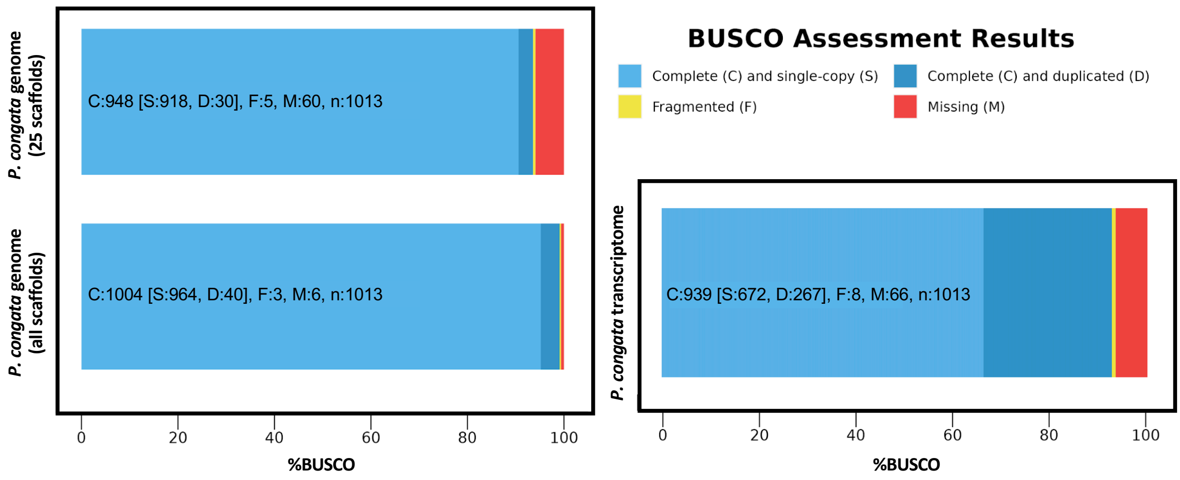

Figure S1: BUSCO assessments of our genome and transcriptome assemblies.

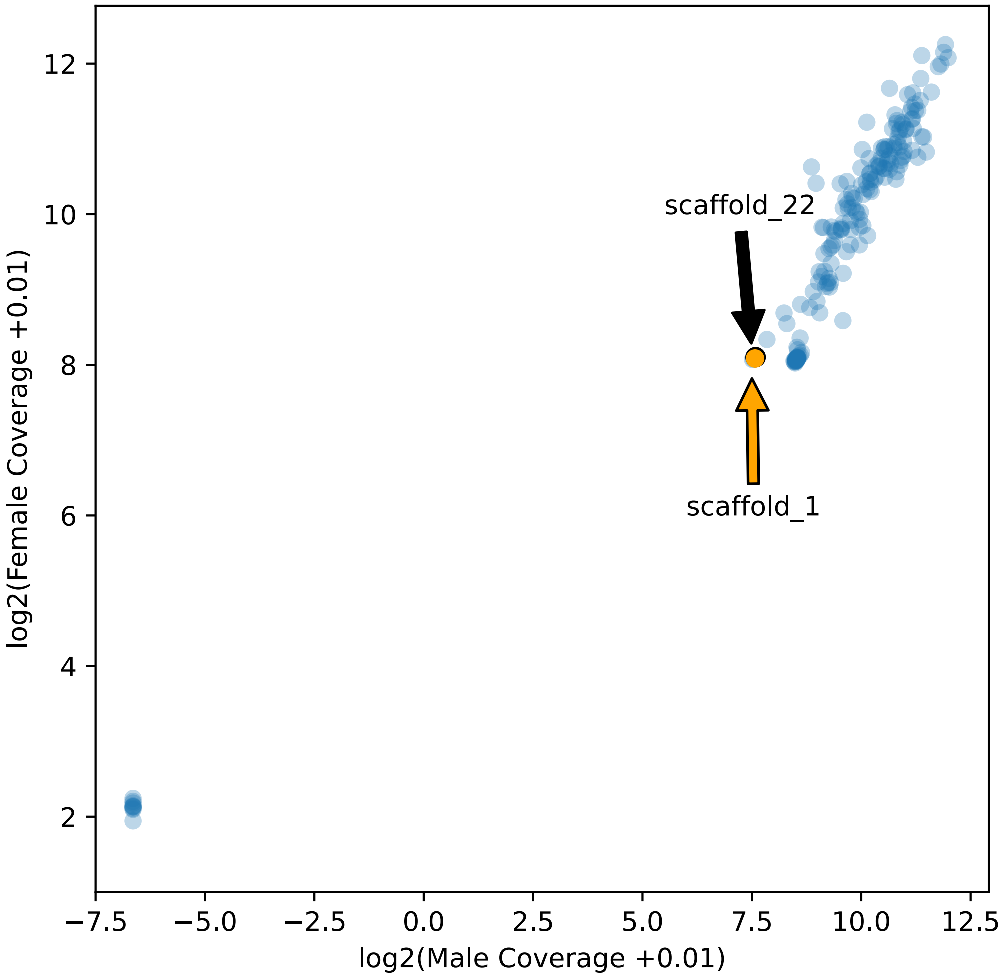

Figure S2: Male and female genome coverage for all scaffolds. X-linked scaffolds are shown with arrows.

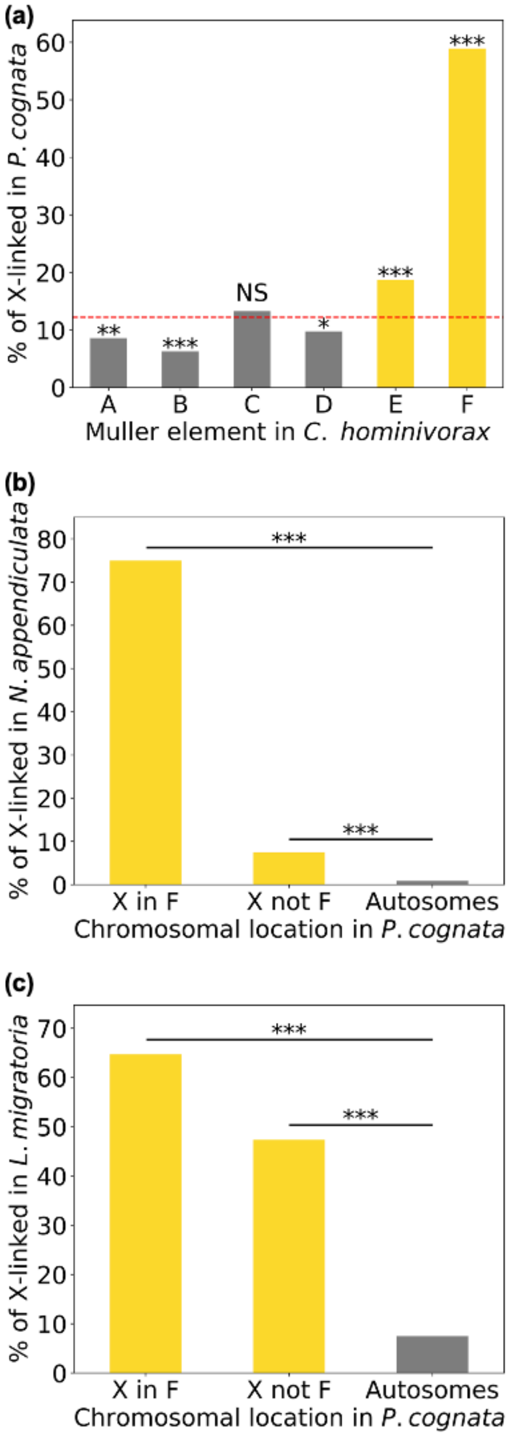

Figure S3: Homology of the X chromosomes of *P. cognata* (order: Mecoptera) and three other insects: two Diptera, *C. hominivorax* (suborder: Brachycera) and *N. appendiculata* (suborder: Nematocera), and *L. migratoria* (order: Orthoptera). All the scaffolds in our P. cognata genome were taken into account. (a) Percentage of genes on each of the C. hominivorax's Muller elements that are X-linked in P. cognata. The red dashed line represents the overall proportion of orthologs that are X-linked in P. cognata (i.e. the "X-linkage threshold"). (b) Percentage of X-linked and autosomal P. cognata genes that are X-linked in N. appendiculata. The X-linked genes of P. cognata were divided into two sets, based on whether they were F-linked in C.hominivorax (X-in-F), or not (X-not-F). (c) same as (b) but showing the percentage of P. cognata genes that are X-linked in L. migratoria. Statistically significant differences between observed and expected percentages were assessed using a chi-squared test (* $P < 0.05$, ** $P < 0.01$, *** $P < 0.001$, NS not significant).

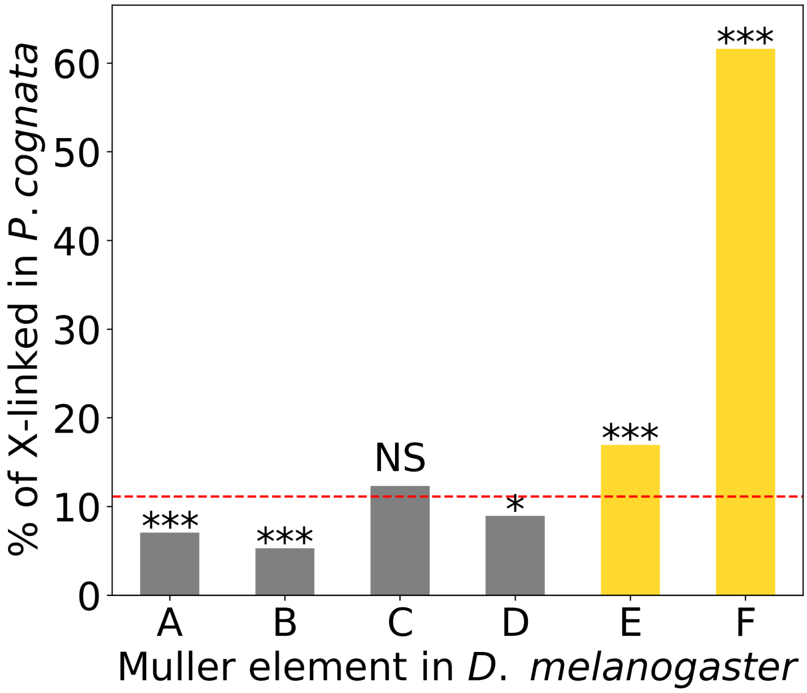

Figure S4: Percentage of genes on each of the *D. melanogaster* Muller elements that are X-linked in *P. cognata.* This is based on 1-to-1 orthologs analysis and the *P. cognata*'s 25 largest genome scaffolds. The red dashed line represents the overall proportion of orthologs that are X-linked in *P. cognata* (i.e. the "X-linkage threshold"). Statistically significant differences between observed and expected percentages were assessed using a chi-squared test (* $P < 0.05$, ** $P < 0.01$, *** $P < 0.001$, NS not significant).

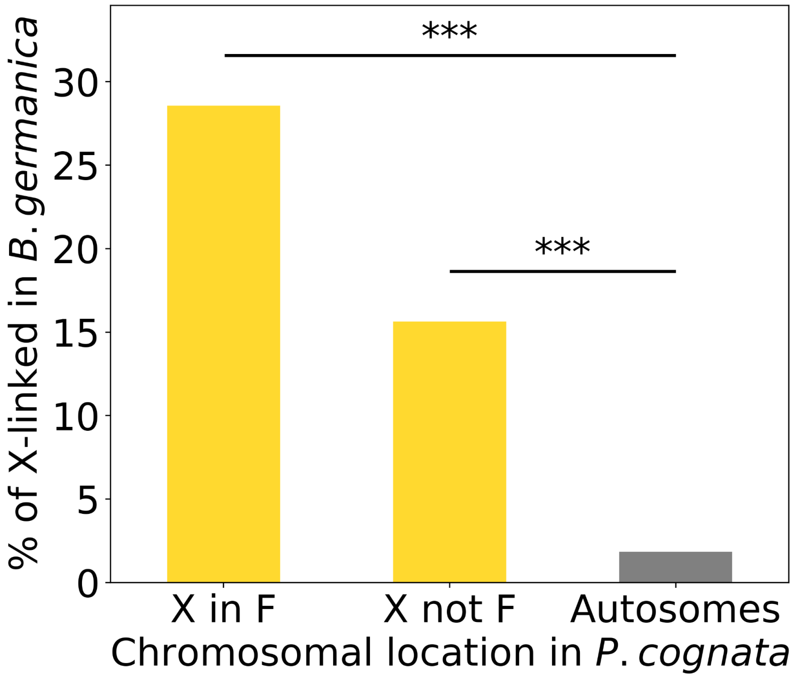

Figure S5: Percentage of X-linked and autosomal *P. cognata* genes that are X-linked in *B. germanica* (order: Blattodea). The X-linked genes of *P. cognata* were divided into two sets, based on whether they were F-linked in *C.hominivorax* (X-in-F), or not (X-not-F). Statistically significant differences between observed and expected percentages were assessed using a chi-squared test (*** $P < 0.001$).

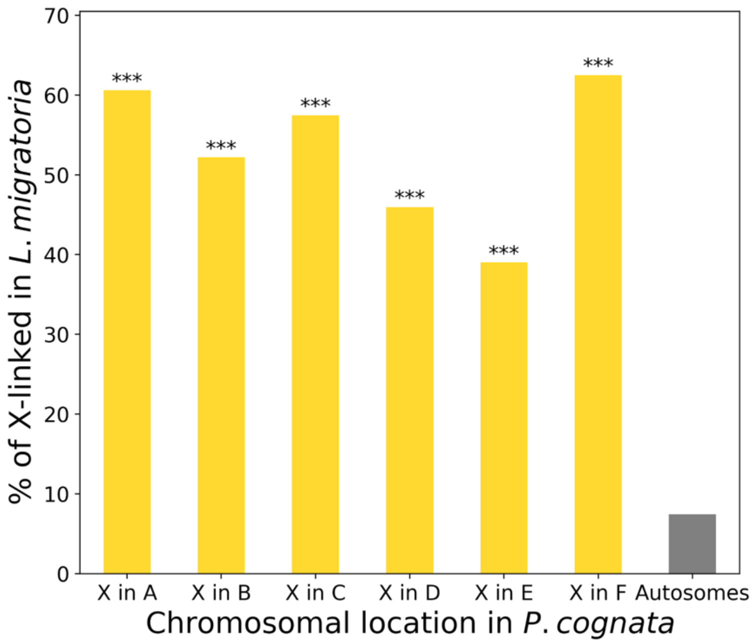

Figure S6: Percentage of X-linked and autosomal *P. cognata* genes that are X-linked in *L. migratoria* (order: Orthoptera). The X-linked genes of *P. cognata* were divided based on their linkage to the 6 Muller elements (A to F) in *C.hominivorax.* Statistically significant differences between each Muller element and the autosomes were inferred using a chi- squared test (*** *P* < 0.001).

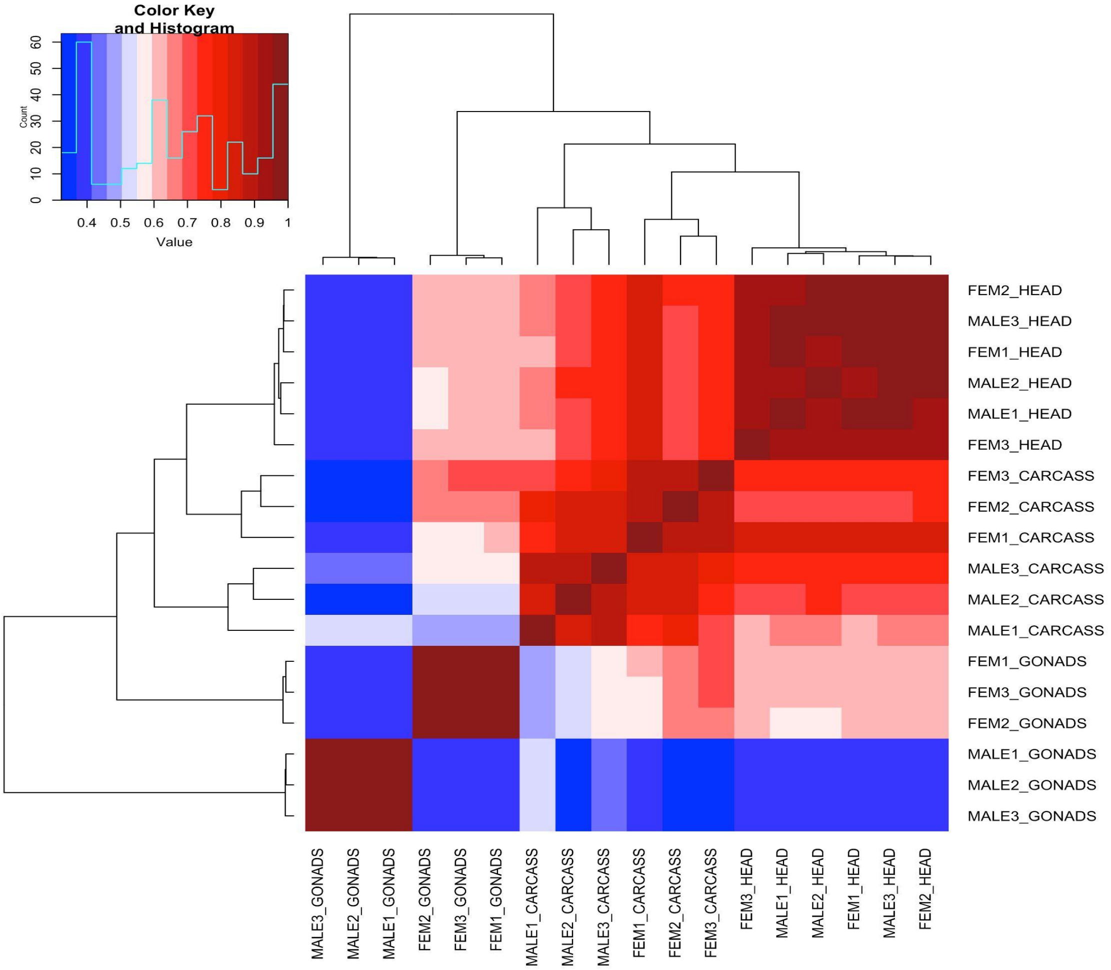

Figure S7: Spearman correlation heatmap displaying the overall similarity in expression profiles of the RNA-seq samples. This is based on the 13785 genes of known chromosomal location. Sample IDs are indicated on the x-axis and secondary y-axis. Similarity between samples is indicated by the dendrogram on the y-axis and the secondary x-axis. Correlation intensity between samples increases from blue (low correlation) to red (high correlation).

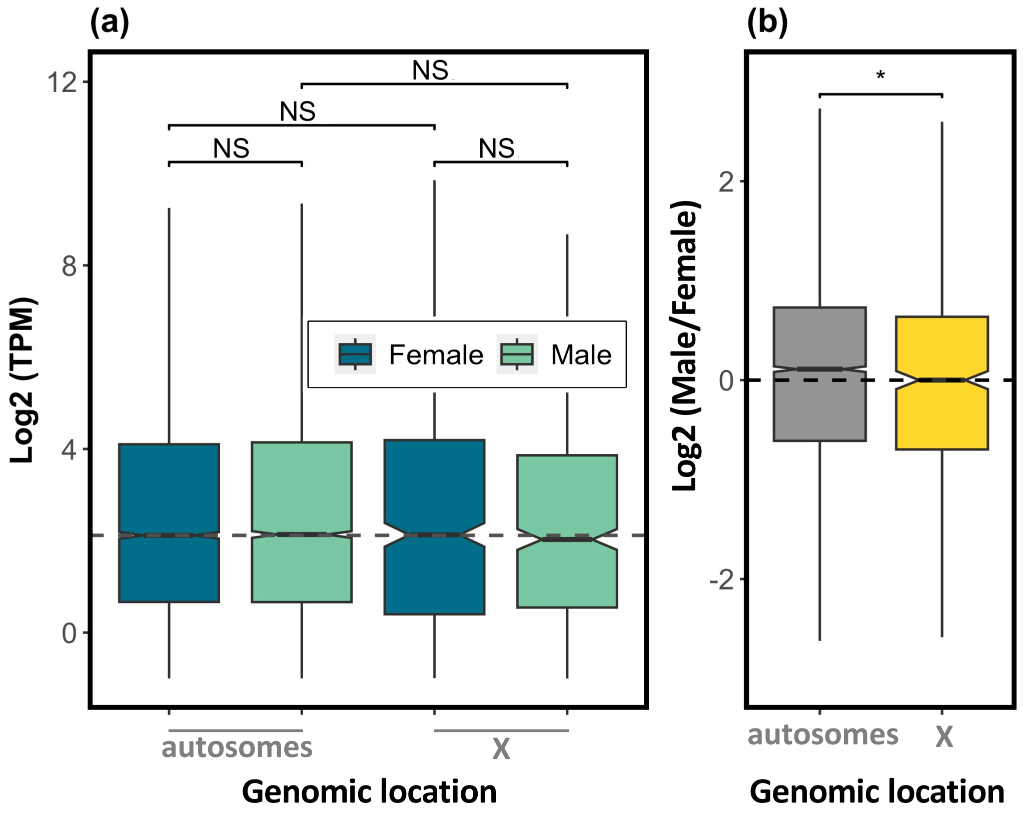

Figure S8: Dosage compensation in carcasses. (a) Expression of autosomal and X-linked genes in males and females (grey dashed line is the female autosomal gene expression median). (b) Log_2 of male-over-female expression ratios for the autosomal and X-linked genes. Statistically significant differences between groups were assessed using a Wilcoxon rank sum test (* *P* < 0.05, ** *P* < 0.01, *** *P* < 0.001, NS not significant).

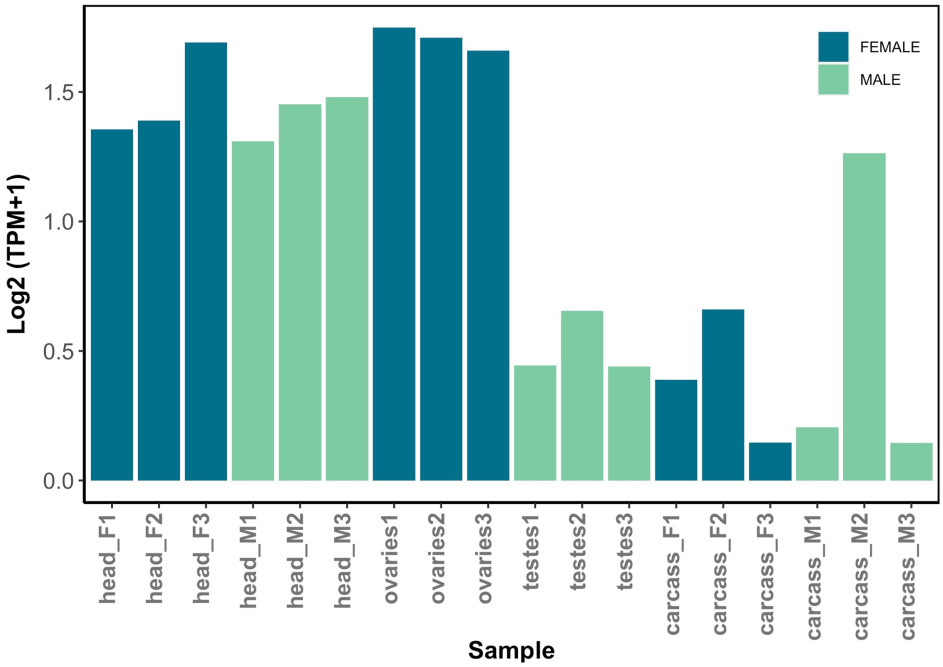

Figure S9: Expression level of gene *Painting Of Fourth* (*POF*) in each RNA-seq sample.

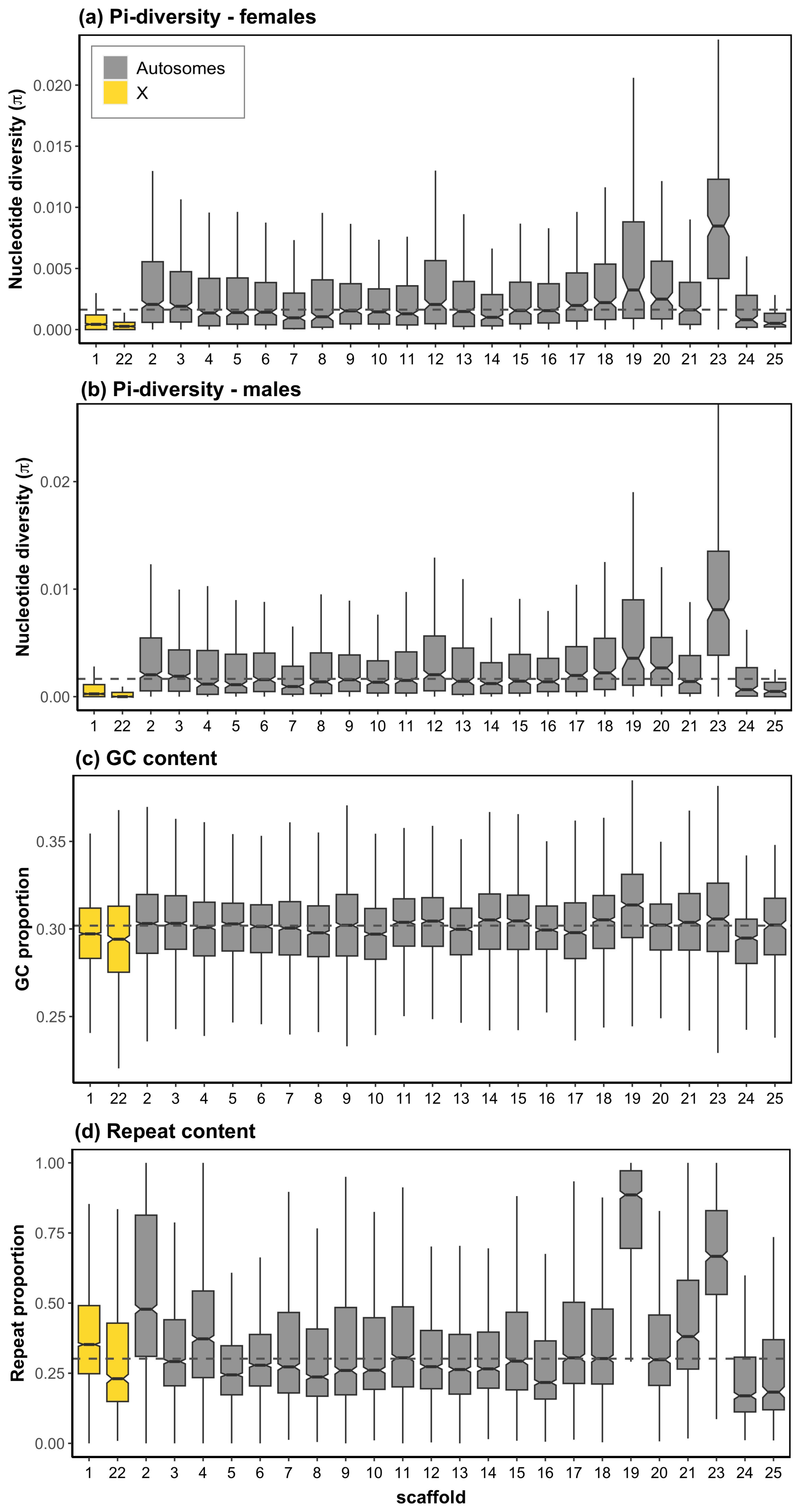

(a) Pi-diversity - females
Autosomes
X
Nucleotide diversity (π)
0.020
0.015
0.010
0.005
0.000
1 22 2 3 4 5 6 7 8 9 10 11 12 13 14 15 16 17 18 19 20 21 23 24 25
(b) Pi-diversity - males
Nucleotide diversity (π)
0.02
0.01
0.00
1 22 2 3 4 5 6 7 8 9 10 11 12 13 14 15 16 17 18 19 20 21 23 24 25
(c) GC content
GC proportion
0.35
0.30
0.25
1 22 2 3 4 5 6 7 8 9 10 11 12 13 14 15 16 17 18 19 20 21 23 24 25
(d) Repeat content
Repeat proportion
1.00
0.75
0.50
0.25
0.00
1 22 2 3 4 5 6 7 8 9 10 11 12 13 14 15 16 17 18 19 20 21 23 24 25
scaffold

Figure S10: Detailed per-scaffold nucleotide diversity, GC and repeat content. (a and b) female and male nucleotide diversity, (c) GC content (per 10000 bp windows) and (d) repeat content (per 10000 bp windows).

Supplementary Tables

Table S1: The classification and distribution of repetitive content on the autosomes and the X chromosome.

	Autosomes (23 scaffolds)			X chromosome (2 scaffolds)		
	#Elements	Length occupied (bp)	% sequence	#Elements	Length occupied (bp)	% sequence
Retroelements	34008	16659493	5.74	4308	1848169	4.28
DNA transposons	47485	10109131	3.49	8945	1844182	4.27
Rolling-circles	6381	1237671	0.43	1216	282311	0.65
Unclassified	440128	79688795	27.48	70177	11944909	27.64
Small RNA	11969	3445476	1.19	1953	619645	1.43
Satellites	338	147685	0.05	18	1729	0
Simple repeats	96244	5095369	1.76	17296	952287	2.2
Low complexity	17721	859362	0.3	2804	136626	0.32

Table S2: List of RNA and DNA samples generated and the analysis they were used for.

Available at : *https://seafile.ist.ac.at/library/b0536613-0190-4733-9f8d-48863df51131/panorpaX/*

Table S3: Statistics for the different assembly steps of the *Panorpa cognata* genome.

	hifiasm	purged	Hi-C scaffolded (scaffolds longer >N80)	Longest 25 scaffolds
size	1034432113	571169746	457046408	333218704
n	6056	1359	187	25
N50	490137	9373981	11774435	13885755

N90	57472	137127	616955	9075028
largest	18224189	18224189	35185269	35185269
Average	170811.12	420286.79	2444098.44	13328748.16
N_count	0	0	2200	1800
Gaps	0	0	11	9

References

Emms DM, Kelly S. 2019. OrthoFinder: phylogenetic orthology inference for comparative genomics. *Genome Biol.* 20:238.

Langmead B, Salzberg SL. 2012. Fast gapped-read alignment with Bowtie 2. *Nat. Methods* 9:357–359.

Meisel RP, Delclos PJ, Wexler JR. 2019. The X chromosome of the German cockroach, *Blattella germanica* , is homologous to a fly X chromosome despite 400 million years divergence. *BMC Biol.* 17:100.
